# OIMHS: An Optical Coherence Tomography Image Dataset Based on Macular Hole Manual Segmentation

**DOI:** 10.1038/s41597-023-02675-1

**Published:** 2023-11-06

**Authors:** Xin Ye, Shucheng He, Xiaxing Zhong, Jiafeng Yu, Shangchao Yang, Yingjiao Shen, Yiqi Chen, Yaqi Wang, Xingru Huang, Lijun Shen

**Affiliations:** 1 Center for Rehabilitation Medicine, Department of Ophthalmology, Zhejiang Provincial People’s Hospital (Affiliated People’s Hospital, Hangzhou Medical College), Hangzhou, Zhejiang China; 2 Wenzhou Medical University, Wenzhou, Zhejiang China; 3 College of Media Engineering, Communication University of Zhejiang, Hangzhou, China; 4 School of Electronic Engineering and Computer Science, Queen Mary University of London, London, UK

**Keywords:** Eye diseases, Biomarkers

## Abstract

Macular holes, one of the most common macular diseases, require timely treatment. The morphological changes on optical coherence tomography (OCT) images provided an opportunity for direct observation of the disease, and accurate segmentation was needed to identify and quantify the lesions. Developments of such algorithms had been obstructed by a lack of high-quality datasets (the OCT images and the corresponding gold standard macular hole segmentation labels), especially for supervised learning-based segmentation algorithms. In such context, we established a large OCT image macular hole segmentation (OIMHS) dataset with 3859 B-scan images of 119 patients, and each image provided four segmentation labels: retina, macular hole, intraretinal cysts, and choroid. This dataset offered an excellent opportunity for investigating the accuracy and reliability of different segmentation algorithms for macular holes and a new research insight into the further development of clinical research for macular diseases, which included the retina, lesions, and choroid in quantitative analyses.

## Background & Summary

A macular hole (MH) is a full-thickness defect of retinal tissue in the fovea of the macula, which can seriously threaten the visual acuity of patients^1^. The overall incidence of MH is approximately 7.8 per 100, 000 individuals per year^2^, with a 64% increased risk in females compared to males^3^. The pathogenesis of MH is not fully clarified, but it is believed to be caused by vitreous traction^4^. Vitrectomy, combined with the internal limiting membrane (ILM) peeling, has been shown to be an effective treatment for MH^5^. A preoperative morphological evaluation of MH and its adjacent structures is beneficial to the selection of the appropriate treatment method and accurate prediction of surgical outcomes.

Optical coherence tomography (OCT), as a non-invasive optical imaging technique, has been rapidly developed during the last decades^6^. OCT has strong tissue penetration, contains stereoscopic imaging, and provides more details of the lesions, thereby supporting the quantification of the lesions directly^7^. Based on OCT images, researchers have proposed a number of predictors of MH surgical efficacy^8^. However, most of these parameters are diameters of MH or intraretinal cysts (IRC), which cannot be used to fully describe the three-dimensional structure of MH. Besides, the measurement process of these parameters is usually implemented manually, which is time-consuming and vulnerable to inter-and intra-measurement errors.

Accurate segmentation of the morphological patterns is crucial for the quantification of the severity of MH, which is helpful in providing imaging biomarkers for the prediction of treatment outcomes. With the introduction of artificial intelligence (AI) to the field of ophthalmology, various AI-based, fully automated models have been developed for lesion segmentation on OCT images, especially for supervised learning-based segmentation algorithms. However, developing an AI-based retinopathy segmentation model requires plenty of annotated retinal images. The training of those models requires large datasets with ground truth segmentation^9^. Manual segmentation by a trained ophthalmologist is the most reliable and is considered as the ground truth segmentation. However, this arduous task is highly time-consuming. Furthermore, the scarcity of public datasets hinders model development. In the currently published database, the OCT data sets are few, and most of the publicly available datasets are those of fundus color photos^10^. These publicly available OCT data sets are summarized in Table 1.

**Table 1:** Summarization of publicly available OCT datasets.

Author	Year	Number of B-scans	Disease	Dataset type
Fang *et al*.^16^	2012	51	AMD, and healthy eyes	Denoised images
Chiu *et al*.^17^	2012	220	AMD	Segmented images of retinal pigment epithelium (RPE), RPE drusen complex, and total retina
Golabbakhsh *et al*.^18^	2013	44	VarIoUs retinal diseases	Original images
Fang *et al*.^19^	2013	195	Healthy eyes	Denoised and interpolated images
Farsiu *et al*.^20^	2014	38400	AMD, and healthy eyes	Segmented images of RPE, RPE drusen complex, and total retina.
Srinivasan.^21^	2014	3231	DR, AMD, and healthy eyes	Images labeled with disease
Chiu *et al*.^22^	2015	110	DR	Segmented images of intraretinal fluid (IRF)
Rashno *et al*.^23^	2017	600	AMD	Segmented images of IRF and subretinal fluid (SRF).
Kermany *et al*.^24^	2018	109312	DR, AMD, and healthy eyes	Images labeled with disease
Lu *et al*.^25^	2019	750	DR, AMD, and healthy eyes	Segmented images of IRF, SRF, and pigment epithelial detachment (PED).
Maetschke *et al*.^26^	2019	1100	Glaucoma	Images labeled with disease
Gholami.^27^	2020	470	DR, AMD, MH, Central serous retinopathy, and healthy eyes	Images labeled with disease

There are some limitations of these datasets. First, those datasets with segmentations are mainly for age-related macular degeneration (AMD) and diabetic retinopathy (DR), and there are no publicly available data sets for MH segmentation. Second, most publicly available OCT image datasets are without segmentation of lesions, and it is very challenging to manually delineate the morphological features of MH. Thirdly, the dearth of datasets assessing the quality of OCT images threatens the accuracy of segmentation techniques. Conducting incorporating assessment of OCT image quality is necessary for improving the performance of segmentation algorithms.

Based on these concerns, we established a large OCT dataset with 3859 images, including manual segmentation of the retina, MH, IRC, and choroid. The segmentation was performed by 3 junior ophthalmologists and corrected by 1 expert ophthalmologist. In addition, the quality of OCT images was evaluated from one objective perspective (i.e., low signal strength) and two subjective perspectives (i.e., signal shield and image blur). Besides the OCT images, this study also provided text data regarding a patient’s status, such as age, sex, and the classification of macular holes, which could support diagnosis, medical concept extraction, clinical decision, and risk assessment. Thus, these OCT image data and text data might be conducive to future research on multimodal data. In addition, this dataset can be applicable to the development of a variety of AI-based image segmentation models to facilitate the development of MH and other disease-related clinical studies.

## Methods

### Data collection

**Fig. 1:**
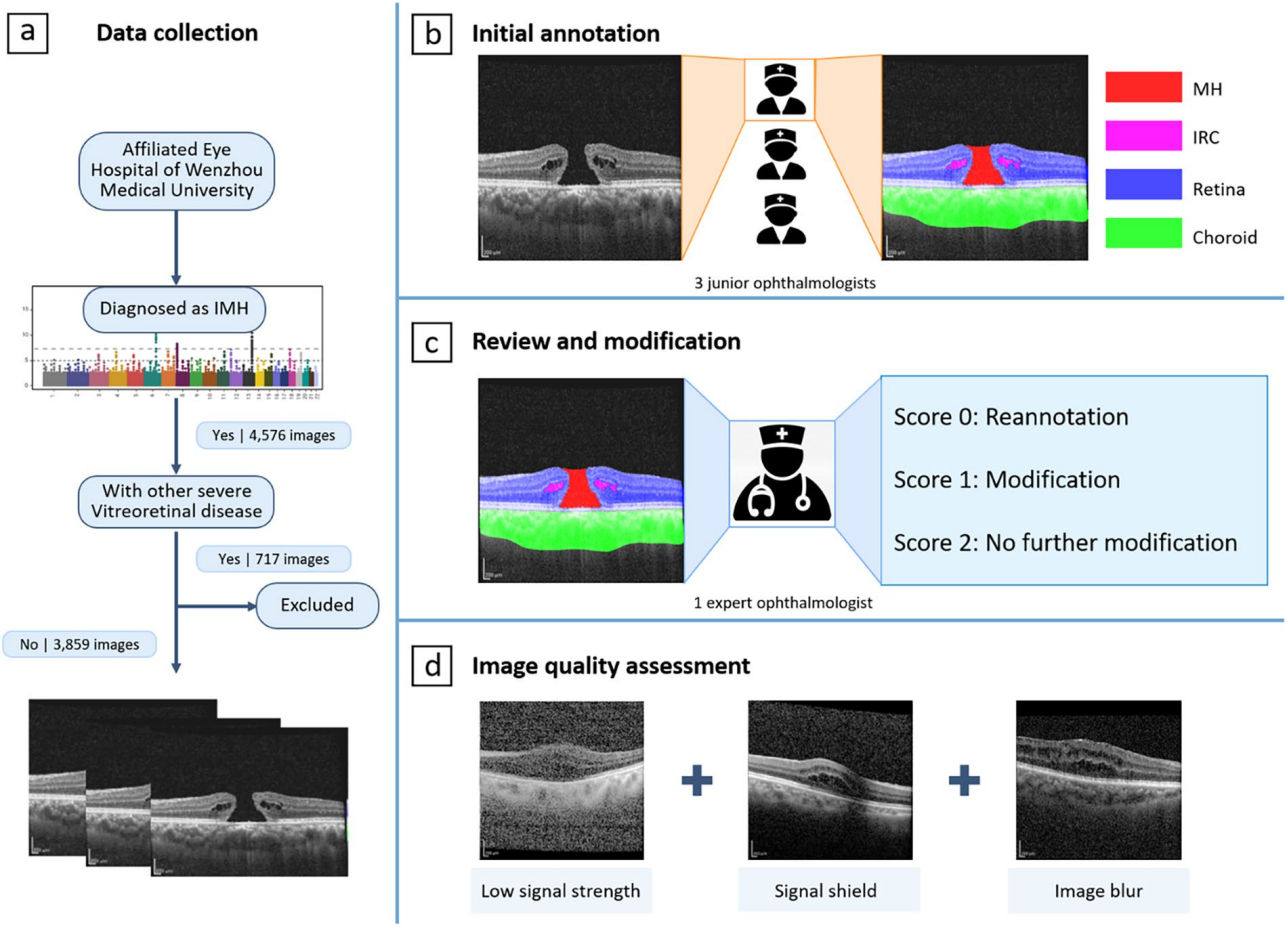
Workflow for establishing the OIMHS dataset. (**a) Data collection**. 3859 OCT B-scan images of 119 patients diagnosed with macular hole were collected in the Affiliated Eye Hospital of Wenzhou Medical University. **(b) Initial annotation**. 3 junior ophthalmologists manually annotated and segmented retina, macular hole, intraretinal cysts, and choroid in the OCT images initially. **(c) Review and modification**. 1 expert ophthalmologist reviewed and verified all the initial segmented images. **(d) Image quality assessment**. The image quality was assessed from three perspectives: low signal strength, signal shield, and image blur.

This retrospective research received approval from the Ethics Committee of Zhejiang Provincial People’s Hospital (QT2023024) and adhered to the tenets of the Declaration of Helsinki. Informed consent was obtained from each patient. A total of 3859 OCT images from 125 eyes of 119 MH patients, who were diagnosed with MH at Zhejiang Provincial People’s Hospital from January 2017 to January 2022, were collected (Fig. 1a). The demographics of the participants were shown in the uploaded Excel file.

OCT volume scans were performed in all patients using the SD-OCT system (Spectralis HRA OCT, Heidelberg Engineering, Heidelberg, Germany). The images were all collected by experienced ophthalmologists. During the scan, the patient was in a sitting position, his/her lower jaw was placed on the jaw brace, and, if necessary, dilated pupils were performed. The scanning procedure required the patient to have a good fixation to ensure that the scanning range was centered on the fovea. Eyes with the following conditions were excluded from this study: (1) previous vitreoretinal surgery; (2) severe vitreoretinal diseases other than MH, such as retinal detachment, high myopic maculopathy, proliferative diabetic retinopathy, retinal artery and vein occlusion, retinitis pigmentosa, central serous chorioretinopathy, glaucoma, retinitis, optic nerve diseases, etc.

### Image annotation: Manually delineation for lesion atlas on the OCT images

The OCT image segmentation annotation group consisted of 3 junior ophthalmologists and 1 expert ophthalmologist (with more than 10 years of working experience). Firstly, the 3 junior ophthalmologists made the initial annotation of all the images. Secondly, the expert ophthalmologist reviewed and corrected all the annotated images. Figure 1 shows the whole workflow of the segmentation annotation process.

During the first step of the initial annotation (Fig. 1b), 3 junior ophthalmologists all used Clip Studio Paint software to manually delineate four anatomical structures (i.e., retina, MH, IRC, and choroid). A short note on the use of Clip Studio Paint software in marking annotations was shown in Supplementary Figure S1. Following were the annotation protocols: (1) The retina was defined as a tissue between the ILM and the retinal pigment epithelium (RPE). (2) MH was defined as a full-thickness defect of retinal tissue in the fovea of the macula, accompanied by exposure to RPE, and presented a low reflex consistent with the vitreous cavity. To determine the inner surface of MH, the annotator first identified the inner surface of the retina on both sides of MH and then bridged the two surfaces to form the inner surface of MH^11^. (3) IRCs were defined as cystoid spaces with low reflexes compared to peripheral retinal tissue. The small IRC away from the MH should be annotated with caution. (4) The choroid is defined as the tissue between the Bruch’s membrane and the sclera. In some images with poor quality, the morphology of tissue on OCT images is incomplete due to noise, shadow artifacts, and other reasons. To minimize false positive annotations and ensure the accuracy of segmentation, junior ophthalmologists were instructed not to modify the annotated pixels solely for the purpose of preserving the original continuity of the anatomic structure. The final OCT image segmentation ground truth was a four-color image, with red pixels representing MH, pink pixels representing IRC, blue pixels representing retina, and green pixels representing choroid.

**Fig. 2:**
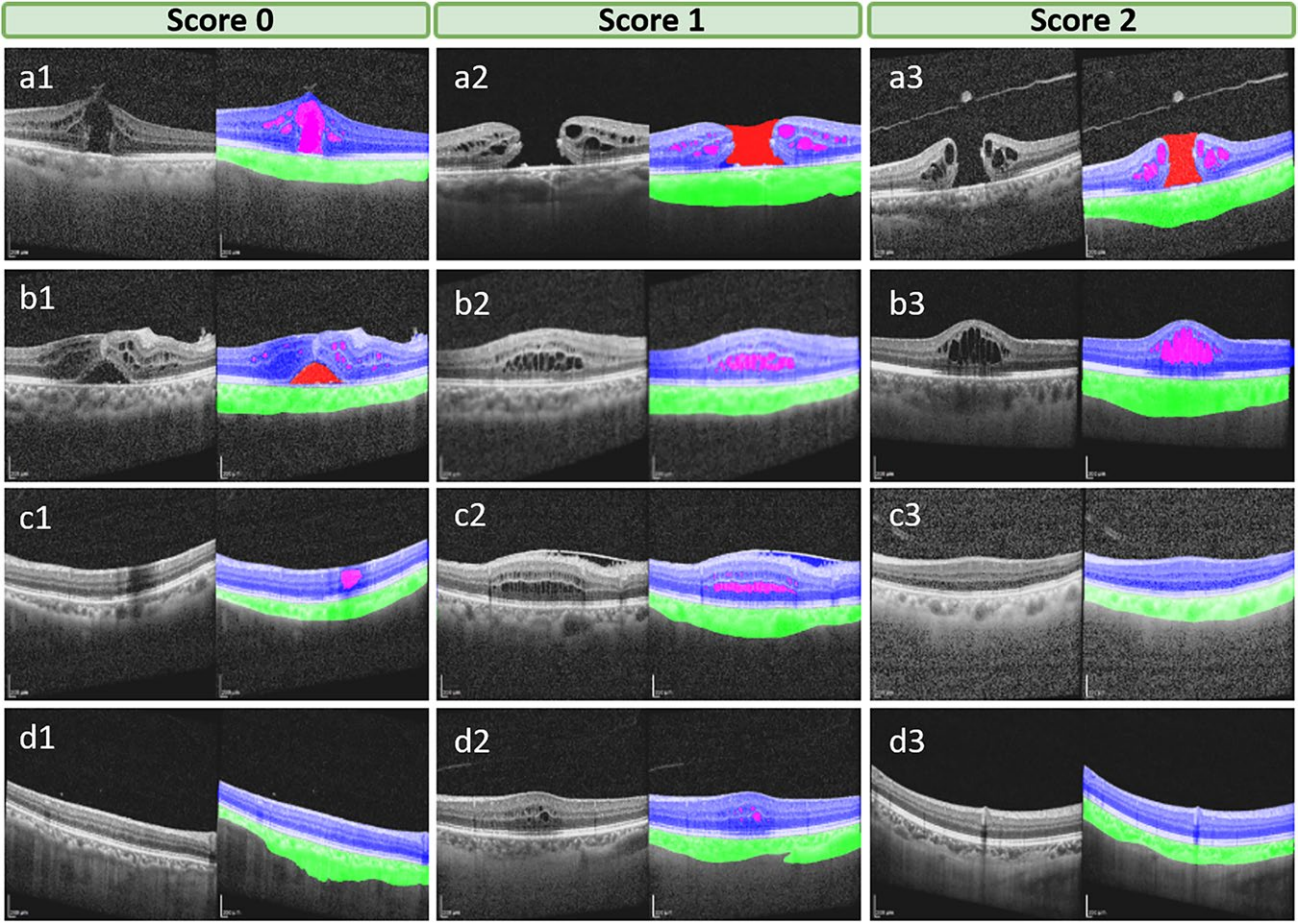
Examples of different scores for the initial annotation. Left column: annotated images with a quality score of 0. middle column: annotated images with a quality score of 1. Right column: annotated images with a quality of 2. The top line list annotated images with different scores for MH annotation; The second line list annotated images with different scores for IRC annotation; The third line list annotated images with different scores for retina annotation; The bottom-line list annotated images with different scores for choroid annotation.

During the second step, all the initial annotated images completed by junior ophthalmologists were reviewed by an expert ophthalmologist who evaluated and scored the quality of annotations and made corresponding decisions (Fig. 1c). The quality of annotations would be scored from 0–2, based on specific scoring criteria. Images in which the four anatomical structures were accurately labeled and segmented were scored as 2 (Fig. 2a3,b3,c3,d3). These annotated images did not need further modification. Images in which the four anatomical structures were accurately labeled but segmented with a few mistakes would be scored as 1 (Fig. 2a2,b2,c2,d2). These annotated images were modified by the expert ophthalmologist using Clip Studio Paint software. Images in which the four anatomical structures were mislabeled and segmented with a wide of mistakes were scored as 0 (Fig. 2a1,b1,c1,d1). These annotated images would be re-annotated by the junior ophthalmologists before the second review by the expert ophthalmologist. During the annotation procession, the expert ophthalmologist would provide junior ophthalmologists with instruction and guidance to improve the accuracy of segmentation.

### Image quality assessment

To account for the substantial impact of various factors on the quality of OCT images and to ensure the usability of the dataset for diverse research applications, we assessed each image in the MH dataset based on one objective image quality perspective (i.e., low signal strength) and two subjective perspectives (i.e., signal shield and image blur) (Fig. 1d). The quality assessment task comprised the evaluation of 3859 images by 3 retinal specialists (each with > 5 years of experience), who utilized a 0.275 mm per pixel monitor positioned at a viewing distance of approximately 30 cm. Any disagreement was adjudicated by the expert retina specialist with over 10 years of work experience. Before we requested their gradings of the images, a generic quality gradation scale (see Table 2) was developed. The scale consisted of three components that together formed a three-bit binary number, generating the following possible grading levels: 000, 001, 010, 011, 100, 101, 110, and 111. Each three-digit set represented an element, with the value of one indicating the agreement between the principal description and the image, and the value of zero representing disagreement. The use of an image quality gradation provided a systematic approach to the investigation of the robustness of an algorithm under varying image quality conditions.

**Table 2:** Quality gradation scale.

Quality perspectives	Low signal strength	Signal shield	Image blur
Grading bits	0/1--	- 0/1 -	--0/1
Principle description	Signal strength ≤ 15	Total or partial loss of retinal and choroid signal	Image has noticeable blur appeared in retina, MH, IRC, and choroid

## Data Records

The OIMHS dataset was uploaded in the form of a zipped file to Figshare^12^. The unzipped file was organized into 1 folder and 2 Microsoft Office Excel lists, named “Images”, “Quality Assessment. xlsx”, and “Demographics of the participants”, respectively. In the “Images” folder, there were 125 subfolders, and each subfolder contains all the images from one individual eye and was named “n”, where “n” represented the ID of an eye. Images were named “n.png”, where “n” meant the number of images. Each image was made up of two parts. The left part was an original OCT image, while the right part was the corresponding ground truth image. In the file “Quality Assessment.xlsx”, there were 5 columns. The first column indicated the ID of an eye. The second column indicated the image name. The third column indicated the low signal strength. The fourth column indicated the signal shield. The last column indicated the image blur. The image quality gradation was either 1 (i.e., good quality) or 0 (i.e., poor quality). In the file “Demographics of the participants”, there were 6 columns. The first column indicated the patient ID. The second column indicated the eye ID. The third column indicated the eye category. The fourth column indicated the age of the patient. The fifth column indicated the sex of the patient and the last column indicated the macular hole stage.

## Technical Validation

### Dataset characteristics

**Fig. 3:**
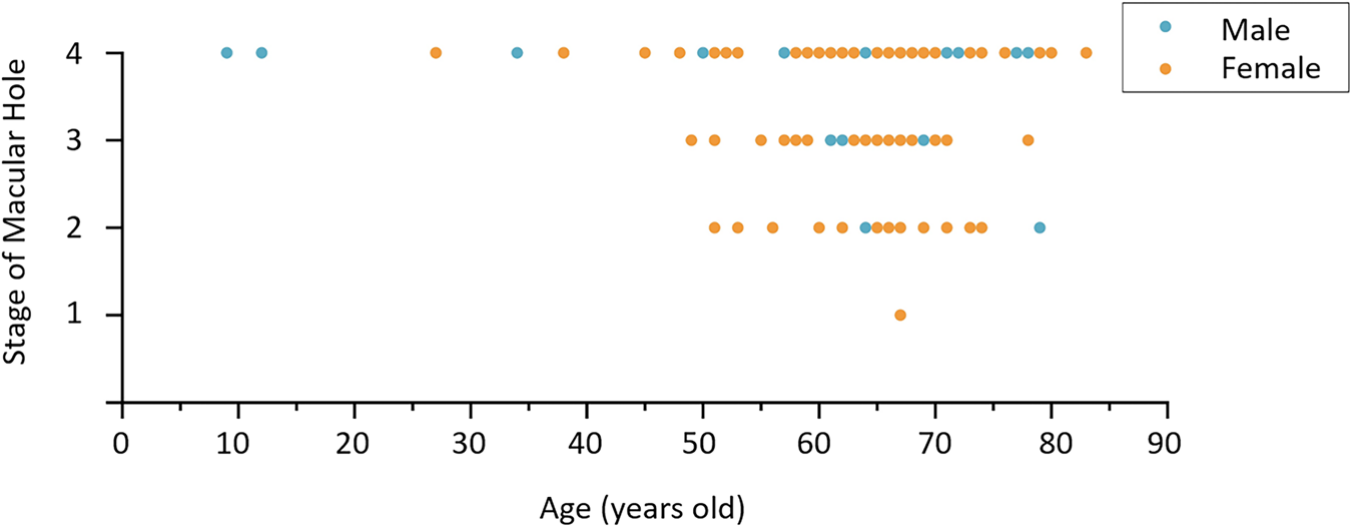
Distribution of the stage of macular hole and the age of patients.

There were 3859 OCT images and their corresponding ground truth images in the dataset. There were 220 images with a resolution of 384 × 496 pixels, 3002 images with a resolution of 512 × 496 pixels, and 637 images with a resolution of 768 × 496 pixels. The key features of the database were summarized in Table 3. These images were from 125 eyes of 119 patients, with 89 females and 30 males. The mean age of the patients was 64.1 years, with a standard deviation of 11.5 years. All the subjects were Asian. Patients were grouped according to the age classification criteria provided by the World Health Organization (WHO). The result showed that there were two teenagers aged below 17, accounting for 0.84% of the sample, three adults aged 18 to 44, accounting for 3.36%, and 22 middle-aged people aged 45 to 59, accounting for 18.49%, and 92 people over 60 years old, accounting for 77.31%. Table 4 shows the distribution of the patients with the specification of gender and age ranges. Based on the Gass classification, MH patients were divided into four stages, and the scatter plot (Fig. 3) shows the distribution of the stage and age of the MH patients.

**Table 3:** Features of the OIMHS dataset.

Particulars	OIMHS dataset
Total number of OCT images	3859
OCT imaging devices used and number of images with different resolutions:	
Heidelberg SD-OCT; image resolution: 384 × 496 pixels	220
Heidelberg SD-OCT; image resolution: 512 × 496 pixels	3002
Heidelberg SD-OCT; image resolution: 768 × 496 pixels	637
Number of ophthalmologists participated in manual annotations	4
Retina ground truth generated out of 3 manual annotations	✓
Macular hole ground truth generated out of 3 manual annotations	✓
Intraretinal cysts ground truth generated out of 3 manual annotations	✓
Choroid ground truth generated out of 3 manual annotations	✓
Images of ground truth were reviewed or modified at least once by expert	✓
Stage of macular hole decision provided by expert	✓

**Table 4:** Distribution of the type of patients in the OIMHS dataset according to gender and age ranges.

Age range (Years old)	Gender	Stage of Macular Hole								Total
		Stage 1		Stage 2		Stage 3		Stage 4		
		RE*	LE**	RE	LE	RE	LE	RE	LE	
<18	Male	0	0	0	0	0	0	2	0	**2**
	Female	0	0	0	0	0	0	0	0	**0**
18–44	Male	0	0	0	0	0	0	0	1	**1**
	Female	0	0	0	0	0	0	2	0	**2**
45–59	Male	0	0	0	0	0	0	0	2	**2**
	Female	0	0	0	3	3	5	8	2	**21**
≥60	Male	0	0	1	2	1	8	7	7	**26**
	Female	0	1	5	5	6	11	21	22	**71**
**Total**		**0**	**1**	**6**	**10**	**10**	**24**	**40**	**34**	**125**

*RE = Right Eye, **LE = Left Eye.

### Image quality distribution

**Fig. 4:**
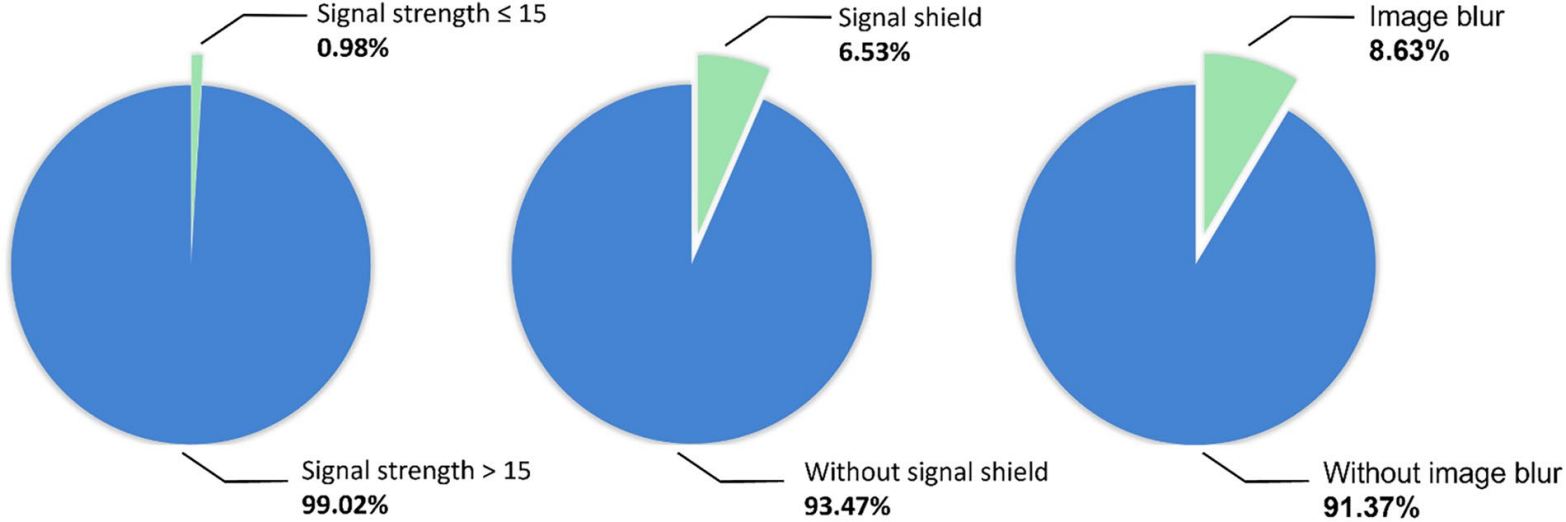
Distribution of different quality images.

All images were evaluated for image quality (see Fig. 4). There were 38 OCT images with the signal strength less than 15, accounting for 0.98% of all collected images, and the signal strength of the other images was all greater than 15. There were 252 OCT images with signal shields, accounting for 6.53%, and the other images were all without signal shields. There were 333 OCT images with image blur, accounting for 8.63%, and the rest were all without image blur. The inclusion of some low-quality images was intended to reflect clinical reality and test the algorithms’ robustness.

### Validation of annotation using quality score

**Fig. 5:**
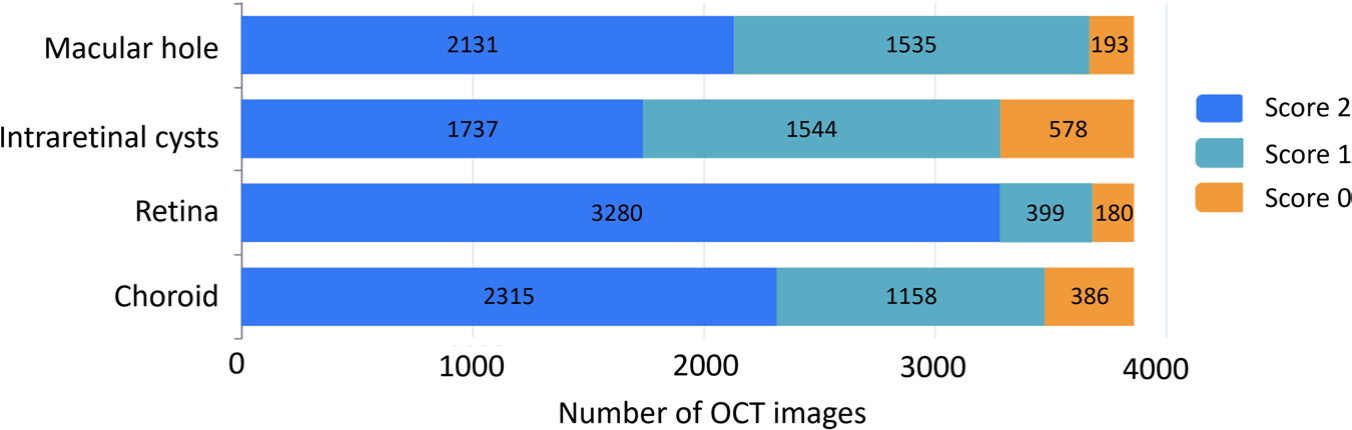
Quality distribution of the initial annotation for four anatomical structures.

Manual annotation is a reliable method for the establishment of the dataset. In this work, every OCT image was initially annotated by 3 junior ophthalmologists and then refined by 1 expert ophthalmologist. The distribution of initial manual annotation quality of all images was counted (Fig. 5). For the initial annotation of MH, 2131 (55.22%) images scored 2, 1535 (39.78%) images scored 1, and 193 (5.00%) images scored 0. For the initial annotation of IRC, 1737 (45.01%) images scored 2, 1544 (40.01%) images scored 1, and 578 (14.98%) images scored 0. For the initial annotation of the retina, 3280 (85.00%) scored 2, 399 (10.34%) images scored 1, and 180 (4.67%) images scored 0. For the initial annotation of the choroid, 2315 (59.99%) images scored 2, 1158 (30.01%) images scored 1, and 386 (10.00%) images scored 0. All the images with a score of 2 were directly included in the dataset, while the images with a score of 1 were modified by the expert ophthalmologist before being added to the database. The images with a score of 0 were re-annotated by the junior ophthalmologists and then reviewed and modified again by the expert ophthalmologist. It took about six minutes to manually annotate an image. The entire image annotation process began in January 2022 and ended in June 2022. All images were reviewed or modified at least once by the expert ophthalmologist. Through the standardized annotation and reviewing process, we established an accurate and reliable OCT dataset based on MH Segmentation.

### Validation of segmentation by evaluating the intra- and inter-consistency of the annotators

To generate a sufficiently reliable dataset, 3 junior ophthalmologists performed manual segmentation of OCT images, and then 1 expert ophthalmologist reviewed and modified the segmentation results. In this study, we used intersection over union (IoU) and the dice coefficient to evaluate the intra- and inter-annotator consistency of the annotators.

**Fig. 6:**
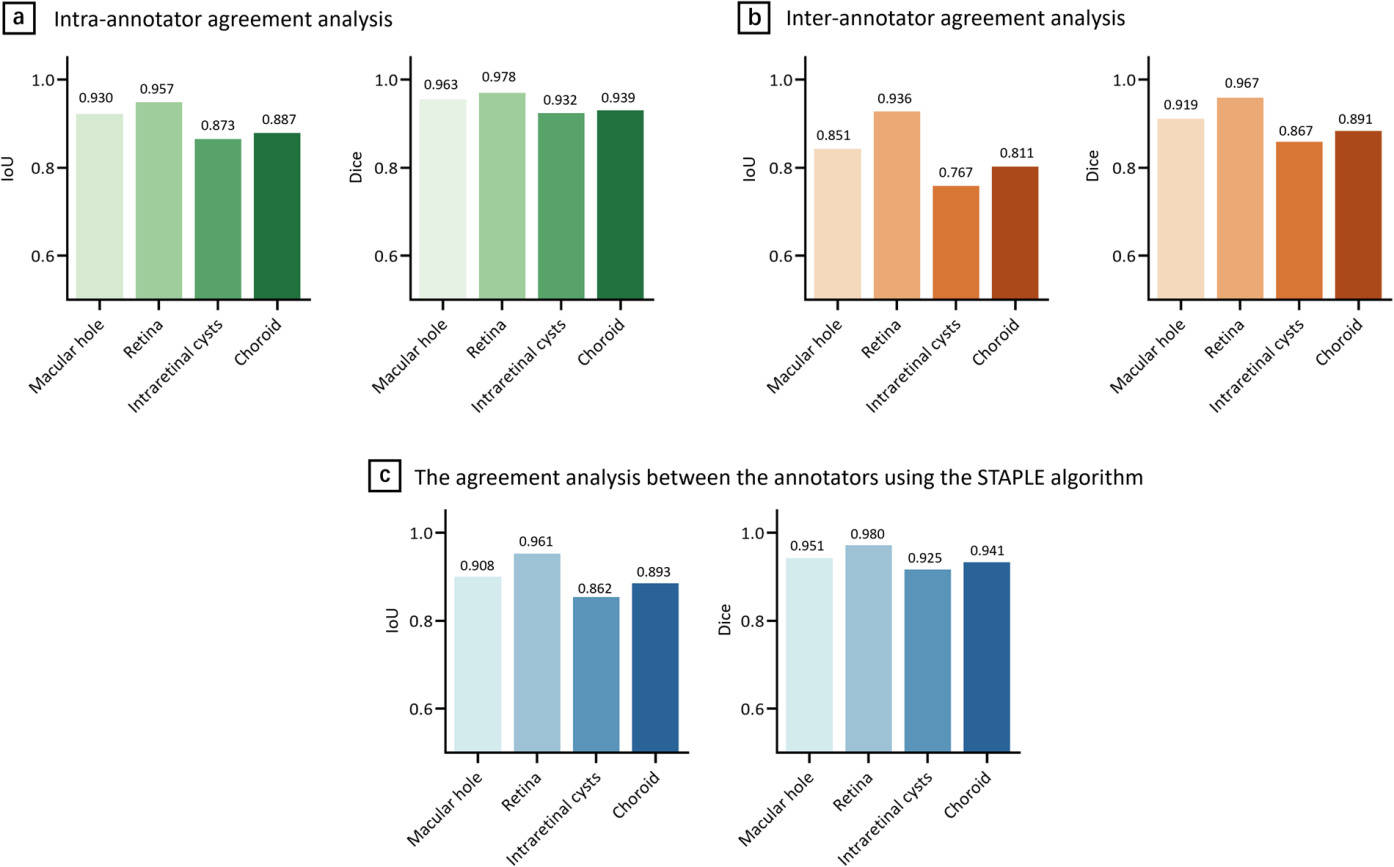
Validation of segmentation. (**a**) **Intra-annotator agreement analysis**. The average IoU for annotation of the four structures was from 0.873 to 0.957. The average Dice coefficient was from 0.932 to 0.978. (**b**) **Inter-annotator agreement analysis**. The average IoU was from 0.767 to 0.936. The average Dice coefficient was from 0.867 to 0.967. (**c**) **The agreement analysis between the annotators using the STAPLE algorithm**. The average IoU was from 0.862 to 0.961. The average Dice coefficient was from 0.925 to 0.980.

For intra-annotator consistency of the same annotator at different times, 20 images of the whole dataset were selected and extracted to form an example set based on the image quality. Three junior ophthalmologists were asked to annotate the 20 images 3 times. IoU and the dice coefficient were calculated for the 1st, 2nd, and 3rd annotations. Mean IoU and the dice coefficient were computed for all 20 images and 3 annotators. For intra-annotator variability of segmentation, the mean IoU and the dice coefficient of MH annotations were 0.930 ± 0.022 and 0.963 ± 0.012, respectively. The mean IoU and the dice coefficient of retina annotations were 0.957 ± 0.015 and 0.978 ± 0.008, respectively. The mean IoU and the dice coefficient of intraretinal cysts annotations were 0.873 ± 0.046 and 0.932 ± 0.026, respectively. The mean IoU and the dice coefficient of choroid annotations were 0.887 ± 0.060 and 0.939 ± 0.036, respectively. These results, presented in Fig. 6 (a) and Supplementary Tables S1, and S2, supported the consistency of the individual annotator in the OCT image segmentation, laying the foundation for accurate annotation and replicable MH OCT image segmentation.

For inter-annotator consistency of the annotators, the error was evaluated using the first annotation of the same 20 images by three junior ophthalmologists. The mean IoU and the dice coefficient of MH annotations were 0.851 ± 0.054 and 0.919 ± 0.031, respectively. The mean IoU and the dice coefficient of retina annotations were 0.936 ± 0.024 and 0.967 ± 0.013, respectively. The mean IoU and the dice coefficient of intraretinal cysts annotations were 0.767 ± 0.067 and 0.867 ± 0.043, respectively. The mean IoU and the dice coefficient of choroid annotations were 0.811 ± 0.107 and 0.891 ± 0.072, respectively. These results, presented in Fig. 6 (b) and Supplementary Tables S3, and S4, supported that the annotation results were close between annotators.

### Validation of segmentation using the STAPLE Algorithm

In order to evaluate the raters’ capacity, the STAPLE algorithm was introduced^13^. The primary objective in integrating the STAPLE algorithm was to comprehensively assess the capacity and reliability of the four raters involved in the labeling process^14,15^. By leveraging this algorithm, we aimed to obtain a robust gold standard annotation encompassing the collective input of three ophthalmologists and one expert. The resulting gold standard serves as a reference point to evaluate the performance and agreement of each rater’s annotation against a more objective and accurate measure. The STAPLE algorithm allowed us to reduce the variability introduced by individual raters during labeling, obtain a more objective and non-biased capacity reference of raters, and further obtain more accurate labels.

An in-depth statistical analysis was conducted to compare the individual raters’ annotations to the gold standard derived through the STAPLE algorithm. The Intersection over Union (IoU) and Dice coefficients metrics were utilized for this comparison. The mean IoU and the dice coefficient of MH annotations were 0.908 ± 0.057 and 0.951 ± 0.032, respectively. The mean IoU and the dice coefficient of retina annotations were0.961 ± 0.025 and 0.980 ± 0.013, respectively. The mean IoU and the dice coefficient of intraretinal cysts annotations were0.862 ± 0.051 and 0.925 ± 0.029, respectively. The mean IoU and the dice coefficient of choroid annotations were 0.893 ± 0.089 and 0.941 ± 0.053, respectively. The results were shown in Fig. 6 (c) and Supplementary Tables S5, and S6. These statistical comparisons shed light on each rater’s proficiency in accurately delineating the macular hole region and other relevant retinal structures. These results provide a clear and insightful assessment of the raters’ capacity to participate in the annotation process and the accuracy of the segmentation, particularly in the critical macular hole region.

## Usage Notes

The whole dataset can be downloaded from the link mentioned above. Users can always split the dataset according to their study design. Users should cite this paper in their research and acknowledge the contribution of this dataset in their study. Users should have received approval from their ethics committee before using this dataset.

### Supplementary information

Supplementary Information

## Data Availability

No novel code was used in the construction of the OIMHS dataset.
