# Supplementary material for: OIMHS: An Optical Coherence Tomography Image Dataset Based on Macular Hole Manual Segmentation
Source: Sci Data. 2023 Nov 6;10:769. doi: 10.1038/s41597-023-02675-1 (PMC10628143; doi:10.1038/s41597-023-02675-1)
Supplement: Supplementary file 1 — Supplementary Information [file 41597_2023_2675_MOESM1_ESM.pdf]

Supplementary Information

Table S1. Intra-annotator Agreement Analysis using IoU

Variables		ophthalmologist 1	ophthalmologist 2	ophthalmologist 3	Average of three ophthalmologists
Macular hole	Attempt 1 - Attempt 2	0.934±0.011	0.950±0.009	0.918±0.026	0.930±0.022
	Attempt 1 - Attempt 3	0.899±0.015	0.948±0.009	0.933±0.009	
	Attempt 2 - Attempt 3	0.919±0.019	0.945±0.014	0.923±0.013	
	Average	0.917±0.021	0.948±0.011	0.925±0.018	
Retina	Attempt 1 - Attempt 2	0.952±0.007	0.970±0.004	0.951±0.008	0.957±0.015
	Attempt 1 - Attempt 3	0.959±0.010	0.968±0.006	0.943±0.021	
	Attempt 2 - Attempt 3	0.953±0.006	0.968±0.007	0.944±0.018	
	Average	0.955±0.008	0.969±0.006	0.946±0.017	
Intraretinal cysts	Attempt 1 - Attempt 2	0.863±0.043	0.892±0.032	0.858±0.045	0.873±0.046
	Attempt 1 - Attempt 3	0.868±0.060	0.899±0.034	0.854±0.041	
	Attempt 2 - Attempt 3	0.864±0.050	0.898±0.035	0.863±0.037	
	Average	0.865±0.051	0.896±0.034	0.859±0.041	
Choroid	Attempt 1 - Attempt 2	0.873±0.025	0.930±0.024	0.906±0.016	0.887±0.060
	Attempt 1 - Attempt 3	0.884±0.039	0.924±0.027	0.837±0.099	
	Attempt 2 - Attempt 3	0.865±0.040	0.913±0.030	0.848±0.088	
	Average	0.874±0.036	0.922±0.028	0.863±0.083	

Table S2. Intra-annotator Agreement Analysis using Dice coefficient

Variables		ophthalmologist 1	ophthalmologist 2	ophthalmologist 3	Average of three ophthalmologists
Macular hole	Attempt 1 - Attempt 2	0.966±0.006	0.974±0.005	0.957±0.014	0.963±0.012
	Attempt 1 - Attempt 3	0.947±0.008	0.973±0.005	0.965±0.005	
	Attempt 2 - Attempt 3	0.957±0.010	0.972±0.008	0.960±0.007	
	Average	0.957±0.011	0.973±0.006	0.961±0.010	
Retina	Attempt 1 - Attempt 2	0.976±0.004	0.985±0.002	0.975±0.004	0.978±0.008
	Attempt 1 - Attempt 3	0.979±0.005	0.984±0.003	0.971±0.011	
	Attempt 2 - Attempt 3	0.976±0.003	0.984±0.004	0.971±0.010	
	Average	0.977±0.004	0.984±0.003	0.972±0.009	
Intraretinal cysts	Attempt 1 - Attempt 2	0.926±0.025	0.943±0.018	0.923±0.026	0.932±0.026
	Attempt 1 - Attempt 3	0.928±0.035	0.946±0.019	0.921±0.024	
	Attempt 2 - Attempt 3	0.926±0.029	0.946±0.020	0.926±0.021	
	Average	0.927±0.030	0.945±0.019	0.923±0.024	
Choroid	Attempt 1 - Attempt 2	0.932±0.014	0.964±0.013	0.950±0.009	0.939±0.036
	Attempt 1 - Attempt 3	0.938±0.022	0.960±0.015	0.908±0.061	
	Attempt 2 - Attempt 3	0.927±0.023	0.954±0.016	0.915±0.052	
	Average	0.932±0.020	0.959±0.015	0.925±0.050	

Table S3. Inter-annotator Agreement Analysis using IoU

		ophthalmologist 1	ophthalmologist 2	ophthalmologist 3	Average
Macular hole	ophthalmologist 1		0.806±0.039	0.900±0.036	0.851±0.054
	ophthalmologist 2	0.806±0.039		0.848±0.037	
	ophthalmologist 3	0.900±0.036	0.848±0.037		
Retina	ophthalmologist 1		0.947±0.017	0.932±0.018	0.936±0.024
	ophthalmologist 2	0.947±0.017		0.929±0.029	
	ophthalmologist 3	0.932±0.018	0.929±0.029		
Intraretinal cysts	ophthalmologist 1		0.789±0.076	0.765±0.056	0.767±0.067
	ophthalmologist 2	0.789±0.076		0.748±0.062	
	ophthalmologist 3	0.765±0.056	0.748±0.062		
Choroid	ophthalmologist 1		0.828±0.078	0.800±0.106	0.811±0.107
	ophthalmologist 2	0.828±0.078		0.805±0.127	
	ophthalmologist 3	0.800±0.106	0.805±0.127		

Table S4. Inter-annotator Agreement Analysis using Dice coefficient

		ophthalmologist 1	ophthalmologist 2	ophthalmologist 3	Average
Macular hole	ophthalmologist 1		0.892±0.024	0.947±0.020	0.919±0.031
	ophthalmologist 2	0.892±0.024		0.917±0.022	
	ophthalmologist 3	0.947±0.020	0.917±0.022		
Retina	ophthalmologist 1		0.973±0.009	0.965±0.010	0.967±0.013
	ophthalmologist 2	0.973±0.009		0.963±0.016	
	ophthalmologist 3	0.965±0.010	0.963±0.016		
Intraretinal cysts	ophthalmologist 1		0.880±0.048	0.866±0.036	0.867±0.043
	ophthalmologist 2	0.880±0.048		0.854±0.041	
	ophthalmologist 3	0.866±0.036	0.854±0.041		
Choroid	ophthalmologist 1		0.904±0.049	0.885±0.070	0.891±0.072
	ophthalmologist 2	0.904±0.049		0.886±0.089	
	ophthalmologist 3	0.885±0.070	0.886±0.089		

Table S5. The Agreement Analysis (IoU coefficient) between the annotator using the STAPLE algorithm

	ophthalmologist 1	ophthalmologist 2	ophthalmologist 3	expert	Average
Macular hole	0.909±0.050	0.878±0.048	0.958±0.027	0.886±0.058	0.908±0.057
Retina	0.951±0.011	0.960±0.016	0.938±0.017	0.996±0.003	0.961±0.025
Intraretinal cysts	0.876±0.047	0.858±0.057	0.845±0.042	0.868±0.049	0.862±0.051
Choroid	0.858±0.058	0.898±0.057	0.832±0.104	0.985±0.021	0.893±0.089
Average	0.899±0.058	0.899±0.061	0.893±0.081	0.934±0.070	0.906±0.070

Table S6. The Agreement Analysis (Dice coefficient) between the annotator using the STAPLE algorithm

	ophthalmologist 1	ophthalmologist 2	ophthalmologist 3	expert	Average
Macular hole	0.951±0.028	0.935±0.027	0.979±0.015	0.938±0.033	0.951±0.032
Retina	0.975±0.006	0.980±0.008	0.968±0.009	0.998±0.002	0.980±0.013
Intraretinal cysts	0.933±0.027	0.923±0.034	0.915±0.025	0.929±0.028	0.925±0.029
Choroid	0.922±0.036	0.945±0.033	0.904±0.068	0.992±0.011	0.941±0.053
Average	0.946±0.033	0.946±0.035	0.942±0.049	0.964±0.038	0.949±0.040

Figure S1. A short note on the use of Clip Studio Paint software in marking annotations. Firstly, an OCT image was imported into the " Clip Studio Paint" software. Secondly, we clicked the “Figure” button and selected the "Bezier Curve". Thirdly, we selected "Spline" and "Create Fill" in the " Tool Properties: Bezier Curve ". For the macular hole, we set the color to RGB (255,0,0) in the “Color slider” and annotated the macular hole boundary. For the retina, we created a new layer by clicking the “New Raster Layer” button, set the color to RGB (0,0,255), and annotated the retina boundary. For the choroid, we set the color to (0,255,0) and annotated the choroid boundary in the same layer. For the intraretinal cysts, we created one more layer and set the color to RGB (255,0,255). After hiding the previous layer, we annotated the intraretinal cysts. Finally, we used “Ctrl+S” to save the image. The details were shown in the below image.

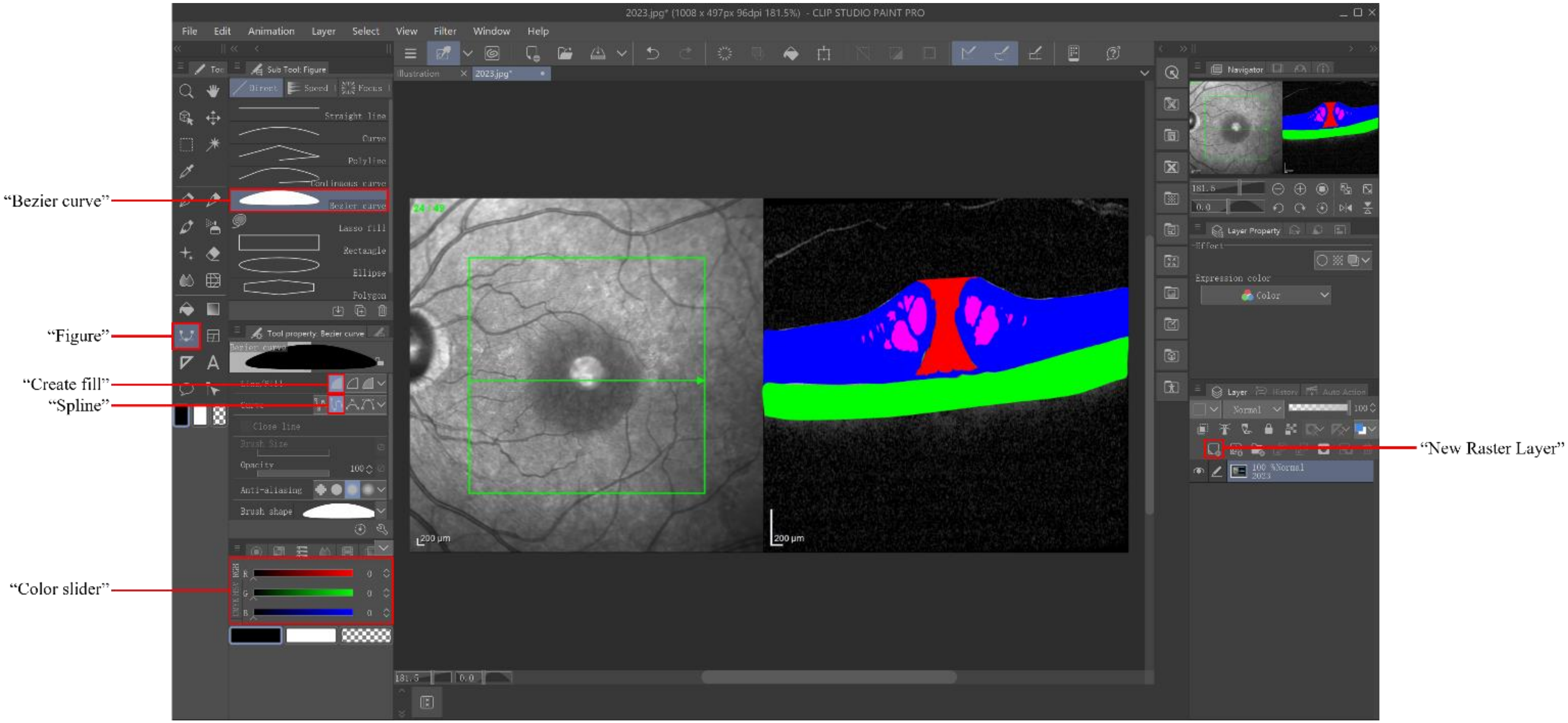

“Bezier curve”
“Figure”
“Create fill”
“Spline”
“Color slider”
“New Raster Layer”
2023.jpg* (1008 x 497px 96dpi 181.5%) - CLIP STUDIO PAINT PRO
File Edit Animation Layer Select View Filter Window Help
200 μm
200 μm
